# TAPI‐1 Combined With Silicone Stents Alleviated Severe Traumatic Tracheal Stenosis via the ADAM17/TGF‐β1 Pathway

**DOI:** 10.1155/carj/9485331

**Published:** 2026-01-11

**Authors:** Zehua Yang, Lan Pan, Tian Xie, Haihong Wu, Kai Liu, Yaqing Li

**Affiliations:** ^1^ Department of Pulmonary and Critical Care Medicine, Hainan General Hospital, Hainan Affiliated Hospital of Hainan Medical University, Haikou, 570311, Hainan, China, hyfyuan.com

**Keywords:** ADAM17, silicone stents, TAPI-1, TGF-β1, traumatic tracheal stenosis

## Abstract

**Background:**

Traumatic tracheal stenosis is characterized by airway granular proliferation after trauma, which may lead to asphyxy. Abnormal activation of the ADAM17/TGF‐β1 pathway may trigger excessive repair and airway fibrosis. TAPI‐1 is an effective inhibitor of ADAM17, which blocks the “extracellular cleavage release” of inflammatory factors (such as TNF‐α) and proliferation signals (such as EGFR ligand TGF‐α) by specifically inhibiting ADAM17 (tumor necrosis factor converting enzyme). Silicone stents are considered an important method for the treatment of airway stenosis. This study aimed to investigate the role and potential mechanism of TAPI‐1 combined with silicone stents in alleviating severe tracheal stenosis after trauma.

**Methods:**

Tracheal epithelial cells were stimulated with GM‐CSF and then treated after 24 h by lentivirus‐mediated ADAM17 RNAi and mitomycin C. A model of severe traumatic tracheal stenosis was established in Beagle dogs. Mitomycin C, TAPI‐1, and TAPI‐1 combined with a silicone stent were applied to treat tracheal stenosis in the animals. The changes in the trachea were directly observed using bronchoscopy. The viability and proliferation of epithelial cells were measured using CCK‐8 assay. The mRNA and protein expressions of ADAM17, TGF‐β1, and fibronectin 1 were detected by qRT‐PCR and western blotting. The pathological changes in traumatic tracheal stenosis were analyzed by hematoxylin and eosin staining.

**Results:**

Lentivirus‐mediated ADAM17 RNAi significantly inhibited the proliferation of tracheal epithelial cells induced by GM‐CSF and suppressed the ADAM17/TGF‐β1 signaling pathway *in vitro*. Moreover, TAPI‐1 combined with silicone stents significantly alleviated traumatic tracheal stenosis in Beagle dogs, and TAPI‐1 markedly inhibited the ADAM17/TGF‐β1 signaling pathway *in vivo*. In conclusion, it was showed that TAPI‐1 combined with silicone stents can significantly alleviate severe traumatic tracheal stenosis in Beagle dogs by inhibiting the ADAM17/TGF‐β1 signaling pathway and expanding the narrowed trachea.

**Conclusions:**

TAPI‐1 combined with silicone stents can be considered as a novel therapeutic approach for treating severe tracheal stenosis after trauma.

## 1. Introduction

Traumatic tracheal stenosis (TTS) is usually caused by granular proliferation and scar formation after tracheal trauma, often presenting as tracheal lumen stenosis, which may lead to dyspnea and asphyxia in severe cases [1]. With the widespread application of tracheal intubation, tracheostomy, and tracheal stents in the treatment of critically ill patients, the incidence rate of TTS is increasing [2]. Patients with TTS have a shorter survival length, a higher risk of long‐term complications, a greater need for psychological support, and lower quality of life. Current treatments mainly focus on surgical or endoscopic interventions [3, 4]. Although surgical operation can be used to resolve granulation and scarring, some patients may suffer from the recurrence of granular proliferation and restenosis after surgery [5]. Endoscopic interventions, characterized by their minimally invasive nature and rapid recovery, are gradually becoming the preferred treatment modality for patients who are not suitable candidates for surgery. However, it also brings challenges, such as postoperative restenosis [6]. Among the various tools utilized in endoscopic intervention, silicone stents are particularly prominent due to their advantages, including plasticity, ease of removal, mild granulation, good tissue compatibility, and a low incidence of complications [7]. Currently, certain anti‐inflammatory and antifibrotic drugs have demonstrated therapeutic efficacy for TTS in experimental settings, although they remain in their infancy [8]. Therefore, exploring more effective treatments with reduced risks of complications is very important. Recently, the rapid advancement of biomedical technology has introduced new solutions for disease management. Inhibitors of some pathways, regarded as innovative therapeutic options, hold significant promise in disease treatment owing to their well‐defined mechanisms of action, strong targeting capabilities, and minimal side effects [9].

The repair process after tissue injury can be divided into four stages: hemostasis, inflammation, proliferation, and maturation/remodeling [10]. Epithelial repair and matrix reconstruction are critical components of the wound healing process, and any abnormalities in these processes can lead to pathological fibrosis and subsequent tracheal stenosis [11]. TGF‐β1 is one of the strongest profibrotic factors, which plays a crucial role in the development of airway fibrosis and airway stenosis [12]. TGF‐β1 is a member of the secretory peptide growth factor superfamily and regulates various biological processes, such as cell proliferation, differentiation, invasion, migration, and apoptosis [13]. TGF‐β1 exacerbates the pathological changes in the airway by affecting the synthesis and degradation of the extracellular matrix [14]. Schäfer and Werner found that continuous administration of TGF‐β1 in animal wound models can induce tissue fibrosis, thereby promoting wound contraction [15]. Wang et al.’s study showed that decreased expression of TGF‐β1 inhibited the proliferation of pathological scars [16]. ADAM17, as a member of the ADAM family (a family of cell surface metalloproteinases), regulates the TGF‐β1 signaling pathway by cleaving the precursor form of TGF‐β1 [17]. Abnormal activation of the ADAM17/TGF‐β1 pathway may change the phenotype and proliferation of airway epithelial cells, thereby triggering excessive repair and fibrosis of the airway [18, 19]. Therefore, inhibiting the ADAM17/TGF‐β1 pathway may provide a new treatment method for TTS. TAPI‐1 is an effective inhibitor of ADAM17, which regulates extracellular matrix remodeling and cell signaling by inhibiting ADAM17. It plays a role in various biological processes [20].

Although TTS may be associated with abnormal repair after injury, the mechanisms underlying this condition remain inadequately understood. TAPI‐1 combined with silicone stents can simultaneously make up for the limitations. In the treatment of complex benign airway stenosis, a complete scheme of immediate relief of obstruction + long‐term prevention of restenosis has been formed. The synergistic advantages are superior to other single treatment methods. This study aimed to investigate the efficacy of TAPI‐1 in combination with silicone stents for alleviating severe TTS by establishing a model of this condition in Beagle dogs. Additionally, this study investigated the effect of ADAM17‐short hairpin RNA (shRNA) on the proliferation of bronchial epithelial cells. The therapeutic effects and mechanisms of TAPI‐1 combined with silicone stents in severe TTS were investigated in this study.

## 2. Materials and Methods

### 2.1. Cell Culture and Recombinant Lentivirus Packaging

Human tracheal epithelial (16HBE) cells were purchased from the Chinese Academy of Sciences (Shanghai, China) and were cultured in RPMI‐1640 medium (Wuhan, China) supplemented with 10% fetal bovine serum and antibiotics (1% penicillin/streptomycin). PCL and silicone stent materials (Xi’an, China) were cut and laid flat at the culture plates. The cells were cultured in a humidified 5% CO_2_ incubator at 37°C. The cells were processed when they reached 80% confluence. GM‐CSF (rHuGM‐CSF, Sangon, Shanghai, China) was dissolved in RPMI‐1640 to prepare a stock solution of 5 ng/mL. Mitomycin (Shanghai, China) was also dissolved in RPMI‐1640. The human ADAM17 mRNA sequence was used to determine suitable siRNA target sequences. The siRNAs were converted into shRNA with a stem‐loop‐stem conformation, and then the recombinant vectors were transformed into DH5a‐competent *Escherichia coli* cells (Invitrogen, Carlsbad, CA, USA). The recombinant vectors and packaging helper plasmids were co‐transfected into 293 T cells with calcium phosphate. The cultured supernatants were collected and centrifuged at 800 × *g* for 7 min to remove cell debris. Finally, the viral particles, LV‐ADAM17‐shRNA, were stored at −80°C.

### 2.2. Cell Proliferation Assay

Based on the manufacturer’s protocol, the toxicity of TAPI‐1 combined with silicone and the toxicity of mitomycin combined with silicone to 16HBE cells were measured using the CCK‐8 kit (C0042, Beyotime Biotechnology, Shanghai, China). First, the cells in each group were diluted into a cell suspension of 3 × 10^4^ cells, and 100 μl of the cell suspension was added to each well. The cells were cultured in a cell incubator at 37°C and 5% CO_2_ for 8, 24, 36, and 72 h. Then, 10 μl of CCK‐8 solution was added and incubated in a cell incubator at 37°C and 5% CO_2_ for 1 h. After incubation, the plate was left at room temperature for 10 min to equilibrate. The absorbance was read at 450 nm using a spectrophotometer (Thermo Fisher Scientific, USA).

### 2.3. Animal Experiment

Fifteen adult male Beagle dogs (10–12 kg, 12 months old) were purchased from Nanjing Chaimen Biotechnology Co. Ltd. The study had been approved by the Medical Ethics Committee of Hainan Provincial People’s Hospital (Yi Lun Yan [2022] No. 634). The procedures were in accordance with relevant guidelines and regulations (GB/T 35823‐2018). Bronchoscopy examination was conducted before surgery to exclude Beagle dogs with congenital tracheal abnormalities.

The Beagle dogs were randomly divided into groups A, B, C, D, and E, three dogs in each group. The dogs underwent a 12‐hour fasting period (overnight) before surgery, followed by the subcutaneous injection of 0.1 mg/kg atropine 15 min before the anesthesia. Anesthesia was induced through an intramuscular injection of 15 mg/kg Zoletil 50. The absence of superficial reflexes, including eyelash movements, corneal reflexes, and responses to painful stimuli, confirmed that the dog was under anesthesia. After induction, each dog was positioned supine on the experimental table. All dogs were stable throughout the intervention and returned to normal condition after the procedure. Rigid bronchoscope was introduced orally, and argon plasma coagulation (APC) was conducted. The power was set at 30 W, with a flow rate of 1.5 L/min. Annular cautery was applied 4 cm above the carina. Balloon compression (Boston Scientific, Ireland) was administered to the cauterized site three times, with each application lasting 30 s, to establish a model of severe TTS in Beagle dogs.

(1) Group A: After inserting a bronchoscope (Zhuhai Shixin Medical Technology Co., Ltd., China) orally, the tracheal condition of the Beagle dogs was observed without any intervention. (2) Group B: After modeling, the tracheal condition of the Beagle dogs was observed without any intervention. (3) Group C: After modeling, local spraying of 0.8 mg/mL TAPI‐1 (1 mL once a week for 3 weeks) was initiated in the first week, and silicone stent was implanted in the third week using rigid bronchoscopy. (4) Group D: Local spraying of 0.8 mg/mL mitomycin (1 mL once a week for 3 weeks) was initiated in the first week after modeling [21]. (5) Group E: Local spraying of 0.8 mg/mL TAPI‐1 (1 mL once a week for 3 weeks) was initiated in the first week after modeling. Bronchoscopy was conducted to measure the airway condition before modeling, immediately after modeling, and at 1, 2, 3, and 4 weeks after modeling. After 4 weeks, the Beagle dogs were euthanized by exsanguination under anesthesia, and tracheal samples were harvested for subsequent experiments.

### 2.4. Western Blotting

Western blotting was performed according to the manufacturer’s instruction. Total proteins were isolated from treated bronchial epithelial cells and tracheal tissues using PMSF (1:100, Amresco, USA) and RIPA lysis buffer (Beyotime Biotechnology, Shanghai, China). Thereafter, the total protein content was measured using a BCA protein assay kit (Beyotime Biotechnology, Shanghai, China). Equal amounts of protein were subjected to SDS‐PAGE and then transferred onto polyvinylidene fluoride membranes (Millipore, USA). After blocking with 5% skim milk at room temperature for 1 h, the membranes were incubated with the following primary antibodies at 4°C for 12 h: ADAM17 (29948‐1‐AP; Proteintech, USA), TGF‐β1 (21898‐1‐AP; Proteintech, USA), fibronectin (15613‐1‐AP; Proteintech, USA), and β‐actin antibody (D110001‐0100; Sangon, Shanghai, China), all at a dilution of 1:1000. The corresponding secondary antibodies were added and incubated in a shaker incubator at room temperature for 1 h. Finally, the immunoblots were measured using an ECL luminescent kit (Beyotime Biotechnology, Shanghai, China), and the optical density of each target protein band was measured using ImageJ software (NIH).

### 2.5. Postoperative Management

After surgery, the dogs in the five groups were observed for any signs of dyspnea, coughing, or abnormal feeding. Suction was conducted if there was a large amount of secretion. Bronchoscopy was used to observe the changes in the tracheal wall and lumen, and screenshots were captured. The relative area of the tracheal lumen was calculated using the ImageJ software. The degree of stenosis was calculated using the following formula: Degree of stenosis = (*S* − *s*)/*S* × 100%, where “*s*” represents the relative area of the tracheal lumen postoperatively, and “*S*” represents the relative area preoperatively.

### 2.6. Histological Analysis

HE staining was used to evaluate the pathological changes in the tracheal mucosa and assess the degree of luminal stenosis. Tracheal tissues were fixed in 10% formalin for more than 24 h. After dehydration and clearing, the tissues were embedded in paraffin and sectioned, followed by HE staining.

### 2.7. Real‐Time Quantitative PCR

#### 2.7.1. Human Source Samples

Total RNA was extracted using TRIzol reagent (Solarbio, Beijing, China) and subsequently reverse transcribed employing the PrimeScript™ RT reagent Kit (RR037A; TAKARA, Japan). Real‐time quantitative PCR was conducted using SYBR® Premix Ex Taq™ II (Tli RNaseH Plus) on a real‐time PCR instrument (LightCycler® 96, Switzerland). The primer sequence is as follows: ADAM17 (fwd: 5′‐GTG​GAT​GGT​AAA​AAC​GAA​AGC​G‐3′, rev: 5′‐GGC​TAG​AAC​CCT​AGA​GTC​AGG‐3′); TGF‐β1 (fwd: 5′‐GGA​AAT​TGA​GGG​CTT​TCG​CC‐3′, rev: 5′‐CCG​GTA​GTG​AAC​CCG​TTG​AT‐3′); fibronectin 1 (fwd: 5′‐TCT​TCT​GGG​TGG​CAG​TGA​TG‐3′, rev: 5′‐TTC​CCT​GGG​GAT​GTG​ACC​AA‐3′); and GAPDH (fwd: 5′‐GAT​GCC​CCC​ATG​TTC​GTC​AT‐3′, rev: 5′‐GTG​TAG​CCC​AGG​ATG​CCT​TT‐3′).

#### 2.7.2. Animal Source Samples

The total RNA was extracted using TRIzol reagent (Shanghai, Sangon, China), and then the reverse transcription reaction was carried out with prepared oligo(dT) primers (Qingke, Beijing, China) and DEPC‐treated water (Sigma, Germany). Real‐time quantitative PCR was conducted using SYBR® Green Realtime PCR Master Mix (Novoprotein, Suzhou, China) on a real‐time PCR instrument (Donglinchangsheng, Beijing, China). The primer sequence is as follows: ADAM17 (fwd: 5′‐AGAGCC ACCTGACGAGTTTG‐3′, rev: 5′‐TCT​TCC​CCT​CTG​CCC​ATG​TA‐3′); TGF‐β1 (fwd: 5′‐CAG​CAT​GTG​GAG​CTG​TAC​CA‐3′, rev: 5′‐GGC​TGG​AAC​TGA​ACC​CGT​TA‐3′); fibronectin 1 (fwd: 5′‐TGG​AAT​GCC​CCA​GAA​CCA​TC‐3′, rev: 5′‐AAG​CGT​GTC​ACC​TCT​CTG​TG‐3′); and GAPDH (fwd: 5′‐GTC​CCC​ACC​CCC​AAT​GTA​TC‐3′, rev: 5′‐GTG​TAG​CCC​AGG​ATG​CCT​TT‐3′).

### 2.8. Statistical Analysis

The statistical analyses were conducted using SPSS V.26.0 software (SPSS, Inc., Chicago, USA). Experimental data are presented as mean ± standard deviation (mean ± SD). For measurement data meeting normal distribution, one‐way analysis of variance (ANOVA) was conducted to assess differences among different experimental groups. Comparisons between the two groups were conducted using two‐tailed Student’s *t*‐test. For data without normal distribution, the Kruskal–Wallis *H* nonparametric test was employed for intergroup comparisons. The criterion for statistical significance was set at a *p* value less than 0.05.

## 3. Results

### 3.1. TAPI‐1 Combined With Silicone Stents Alleviated Severe TTS in Beagle Dogs

**Figure 1:**
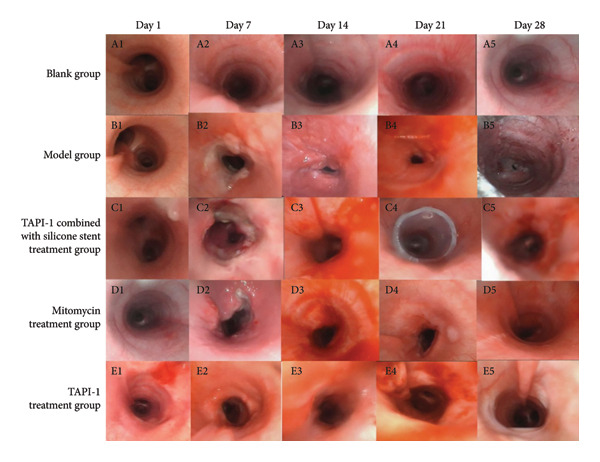
Silicone stent combined with TAPI‐1 alleviates severe traumatic tracheal stenosis. (a) Blank control group. (b) Model control group. (c) TAPI‐1 combined with silicone stent treatment group. (d) Mitomycin treatment group. (e) TAPI‐1 treatment group.

A severe traumatic airway stenosis model was established in Beagle dogs to determine the effects of TAPI‐1 combined with silicone stents on severe traumatic airway stenosis. On days 1, 7, 14, 21, and 28, the tracheal structure was intact under bronchoscopy in the control group, with no abnormal secretions or stenosis in the tracheal lumen, no congestion of the tracheal wall, and intact mucosal surface (Figure 1(a)). Before modeling, the tracheal structure in the model control group was intact with no significant abnormalities (Figure 1(b)). On day 7, white necrotic material appeared at the injury site, and the tracheal wall exhibited noticeable congestion. Granulation was observed on day 14, the necrotic material was absorbed, and tracheal stenosis was established. On day 21, the necrotic material was absorbed, but the granulation tissue continued to proliferate. On day 28, stenosis was obvious. Silicone stent was implanted on day 21, and the degree of airway stenosis was significantly alleviated on day 28 (Figure 1(c)). The degrees of airway stenosis were significantly alleviated by both TAPI‐1 and mitomycin (Figures 1(d) and 1(e)).

The HE staining of the model control group revealed hyperplasia of the airway epithelium and thickening of the submucosa (Figure 2), both of which protruded into the lumen, leading to stenosis. There were increases in fibrous tissue, smooth muscle tissue, small blood vessels, and mucosal glands, along with abundant lymphocytic infiltration in the lamina propria. The thickness of the granulation tissue in the trachea of the Beagle dogs in the model control group was significantly greater than that in the negative control group, suggesting the successful establishment of the severe traumatic airway stenosis model in Beagle dogs. Moreover, TAPI‐1, mitomycin, and the combination of TAPI‐1 with silicone stents all alleviated the pathological changes and stenosis of the trachea.

Figure 2TAPI‐1 combined with silicone stents alleviated the pathological changes in trachea stenosis. (a) Blank control group. (b) Model control group. (c) TAPI‐1 combined with silicone stent treatment group. (d) Mitomycin treatment group. (e) TAPI‐1 treatment group.

**Figure:**
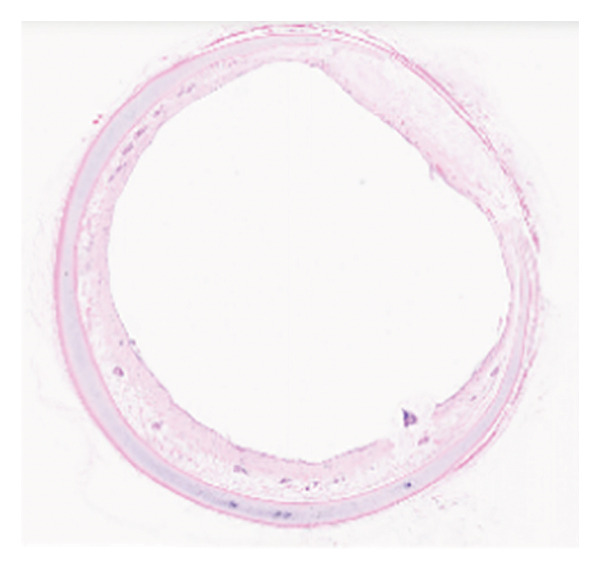
(a)

**Figure:**
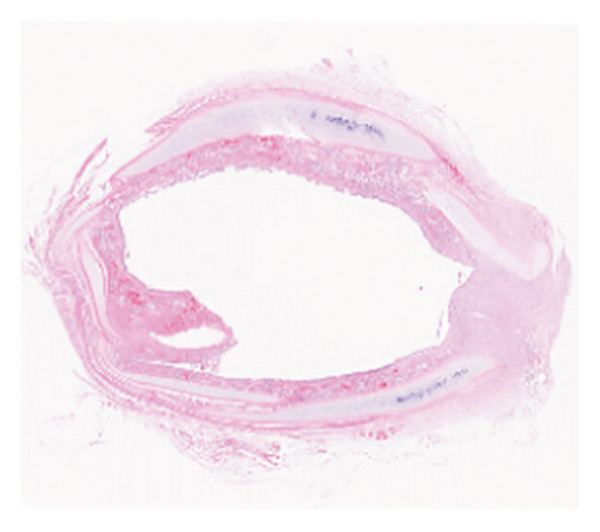
(b)

**Figure:**
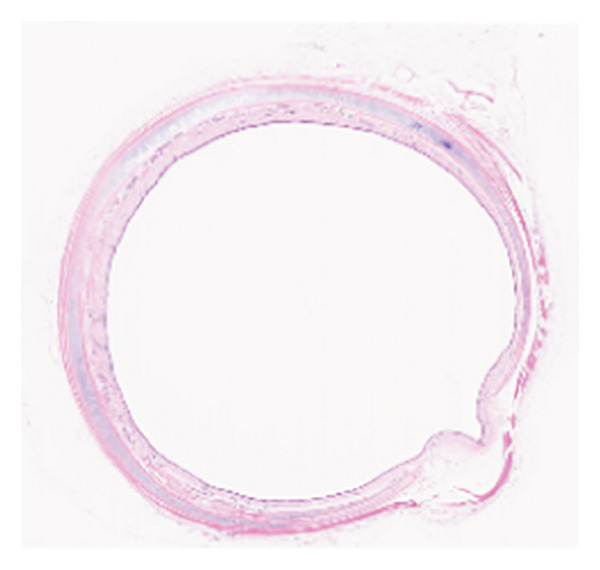
(c)

**Figure:**
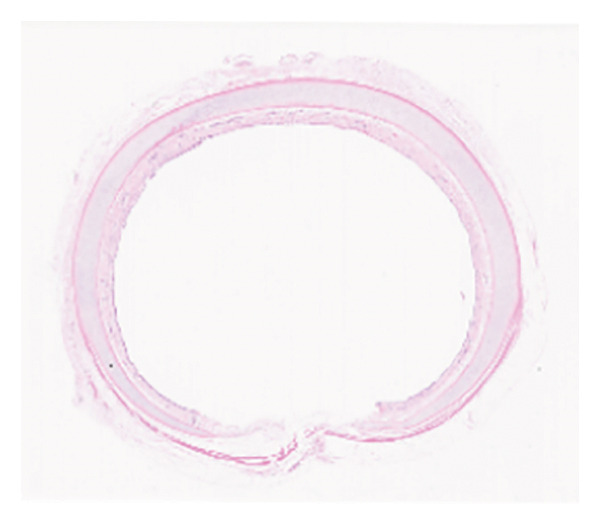
(d)

**Figure:**
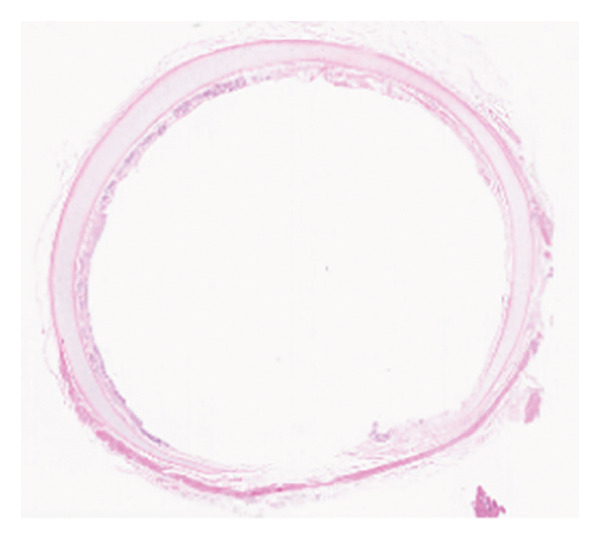
(e)

**Figure 3:**
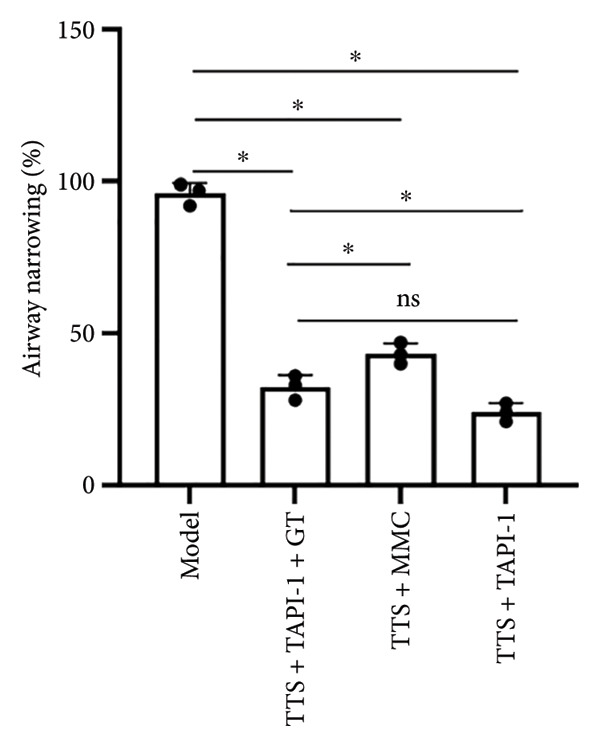
The degree of tracheal stenosis in each group on the 28th day. *n* = 3. ^∗^ *p* < 0.05. ^ns^ *p* > 0.05.

Furthermore, the degree of stenosis in the TAPI‐1 treatment group, mitomycin treatment group, and TAPI‐1 combined with silicone stent treatment group became better than that in the model control group (*p* < 0.05) (Figure 3). Moreover, the TAPI‐1 treatment group had the lowest degree of stenosis, but there was no significant difference between the TAPI‐1 treatment group and the TAPI‐1 combined with the silicone stent treatment group (*p* > 0.05).

### 3.2. The Expressions of TGF‐β1 and Fibronectin 1 Were Downregulated by TAPI‐1, Inhibiting ADAM17/TGF‐β1 Pathway in Trachea Stenosis

The mRNA and protein levels of ADAM17, TGF‐β1, and fibronectin 1 were significantly upregulated in Beagle dogs with severe TTS (Figure 4). The mRNA and protein levels of TGF‐β1 and fibronectin 1 were significantly downregulated after treatment with TAPI‐1 and mitomycin. Mitomycin did not affect the mRNA expression of ADAM17 (*p* > 0.05). Furthermore, compared with those of the TAPI‐1 group or the mitomycin group, the mRNA and protein expressions of ADAM17, TGF‐β1, and fibronectin 1 were significantly increased in the TAPI‐1 combined with silicone stent treatment group (*p* < 0.05), which indicated that the protein expressions of TGF‐β1 and fibronectin 1 were increased due to the stimulation of the silicone stents. Notably, compared to those of the mitomycin group, the mRNA and protein expressions of TGF‐β1 and fibronectin 1 were significantly reduced in the TAPI‐1 group (*p* < 0.05) (Figure 4).

Figure 4The expressions of ADAM17, TGF‐β1, and fibronectin 1 in trachea stenosis. (a–c) The mRNA expressions of ADAM17, TGF‐β1, and fibronectin 1. (d–g) The protein expression of ADAM17, TGF‐β1, and fibronectin 1. *n* = 3. ^∗^ *p* < 0.05. ^∗∗^ *p* < 0.01. ^∗∗∗^ *p* < 0.001. ^∗∗∗∗^ *p* < 0.0001. ^ns^ *p* > 0.05.

**Figure:**
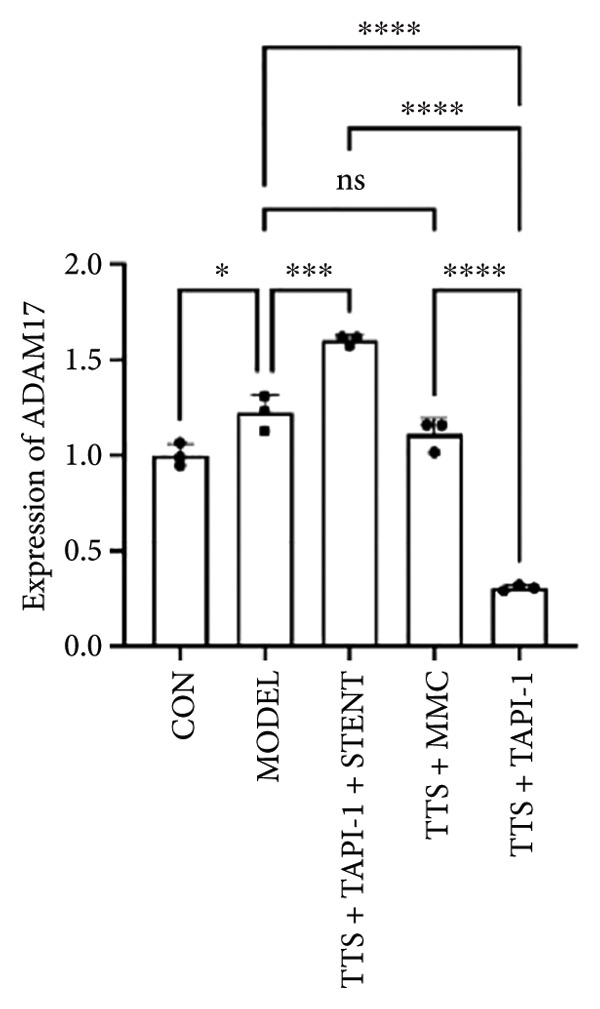
(a)

**Figure:**
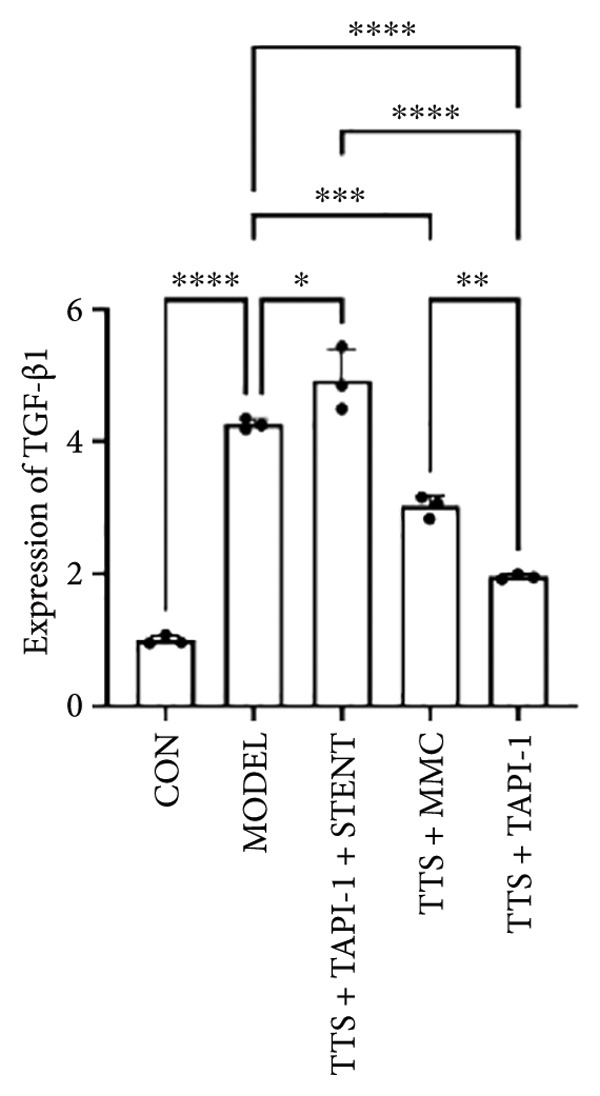
(b)

**Figure:**
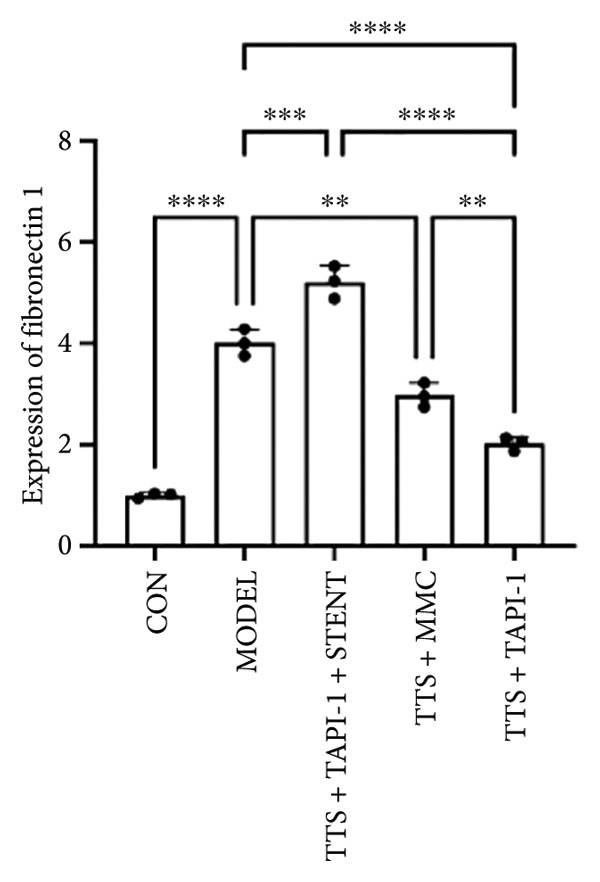
(c)

**Figure:**
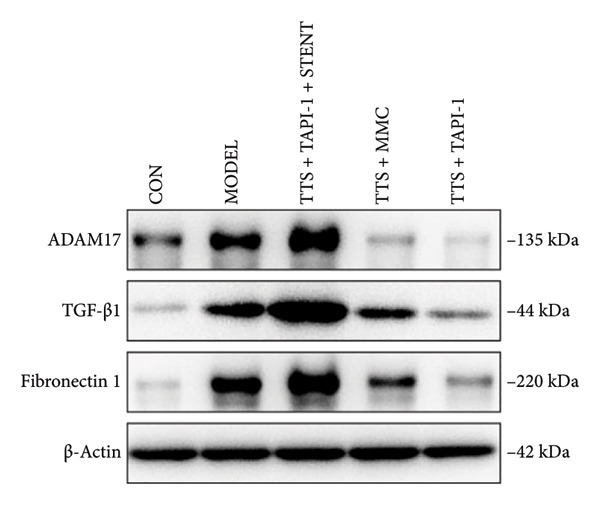
(d)

**Figure:**
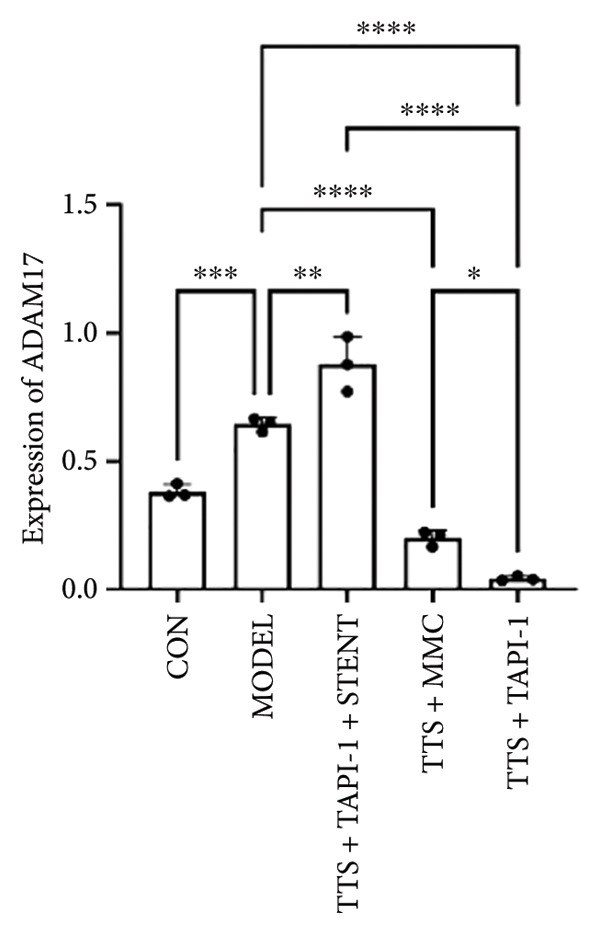
(e)

**Figure:**
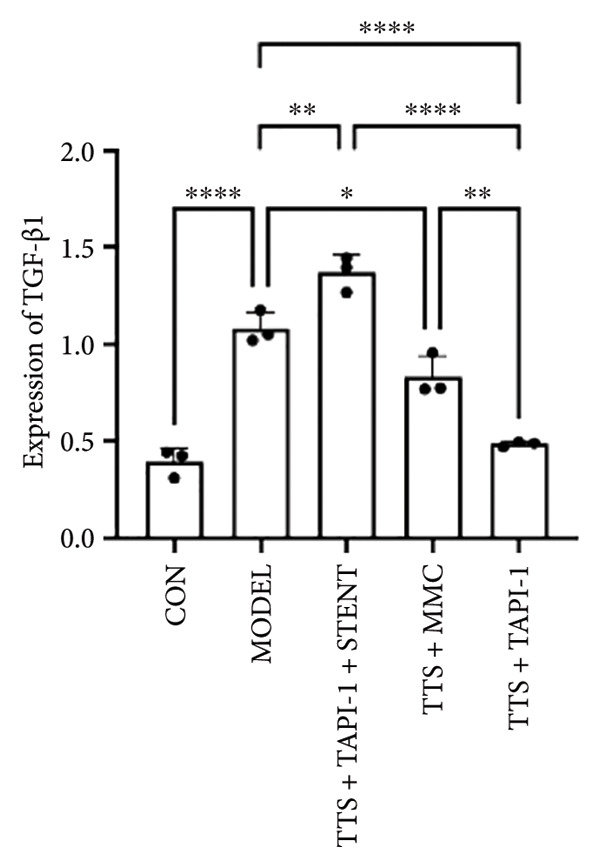
(f)

**Figure:**
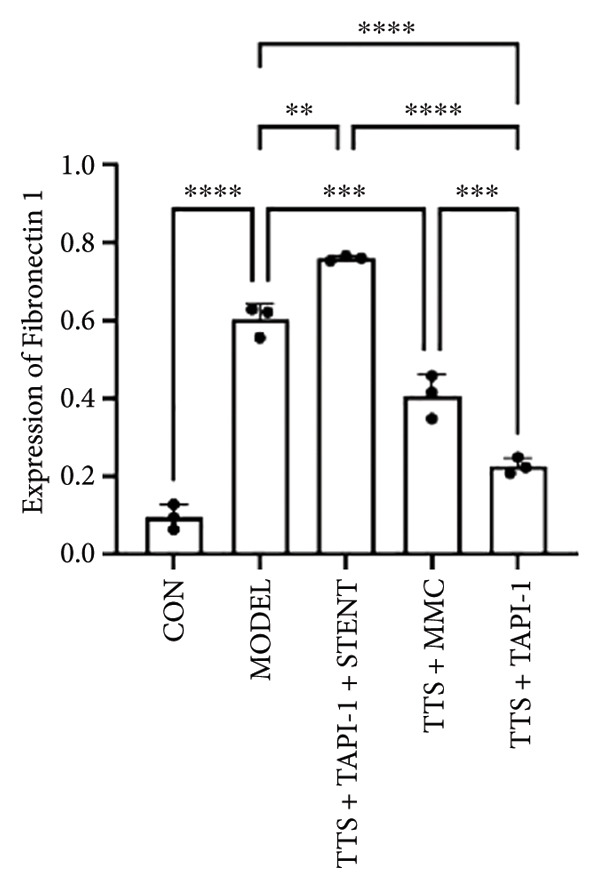
(g)

### 3.3. The Expressions of TGF‐β1 and Fibronectin 1 Were Inhibited by Lentivirus‐Mediated ADAM17 RNAi in Tracheal Epithelial Cells

The mRNA and protein expressions of TGF‐β1 and fibronectin 1 in 16HBE cells induced by GM‐CSF were significantly increased than those of the control group (*p* < 0.0001), and there was no significant difference in the mRNA expression of ADAM17 (*p* > 0.05). Moreover, the mRNA and protein expression levels of TGF‐β1 and fibronectin 1 were significantly increased than those of the model group in the stimulation with silicone material; however, there was no significant difference in the mRNA expression of ADAM17 (*p* > 0.05). Both lentivirus‐mediated ADAM17 RNAi and mitomycin downregulated the expressions of ADAM17, TGF‐β1, and fibronectin 1 (*p* < 0.0001). Thus, it indicated that both lentivirus‐mediated ADAM17 RNAi and mitomycin significantly inhibited the expression levels of ADAM17, TGF‐β1, and fibronectin 1 induced by GM‐CSF (Figure 5).

Figure 5The expressions of TGF‐β1 and fibronectin 1 induced by GM‐CSF were inhibited by lentivirus‐mediated ADAM17 RNAi in tracheal epithelial cells. (a–c) The mRNA expressions of ADAM17, TGF‐β1, and fibronectin 1. (d–g) The protein expressions of ADAM17, TGF‐β1, and fibronectin 1. *n* = 3. ^∗^ *p* < 0.05. ^∗∗^ *p* < 0.01. ^∗∗∗^ *p* < 0.001. ^∗∗∗∗^ *p* < 0.0001. ^ns^ *p* > 0.05.

**Figure:**
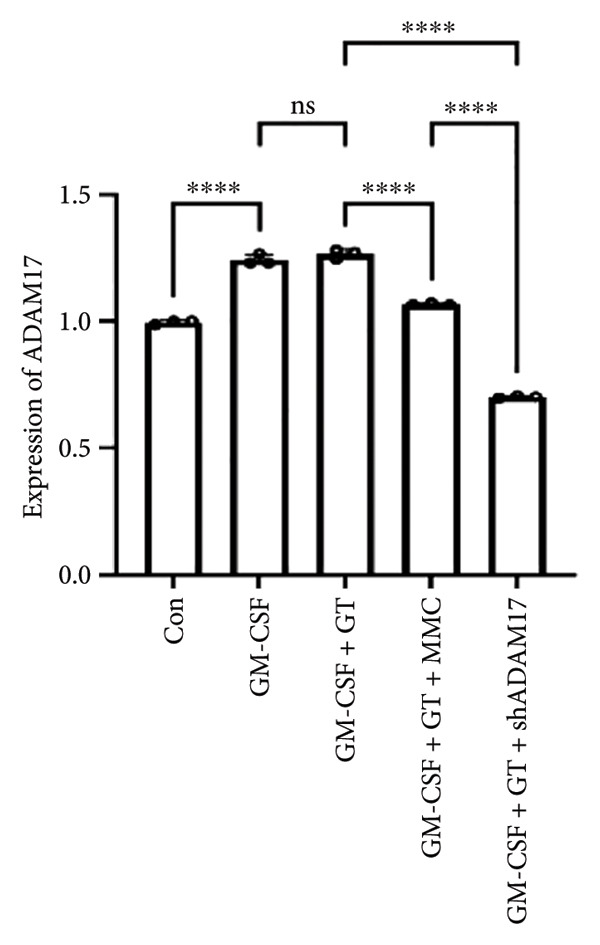
(a)

**Figure:**
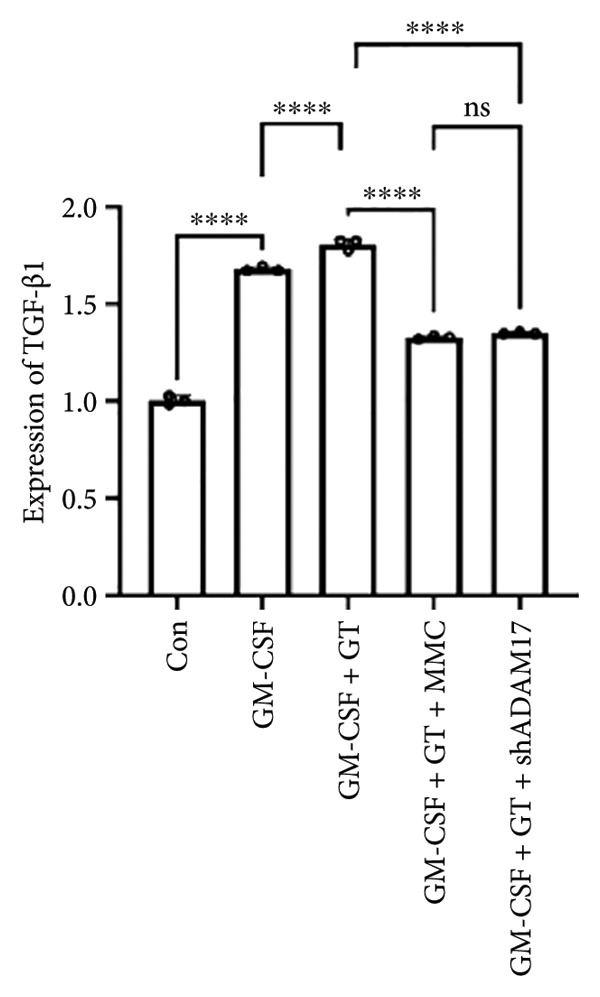
(b)

**Figure:**
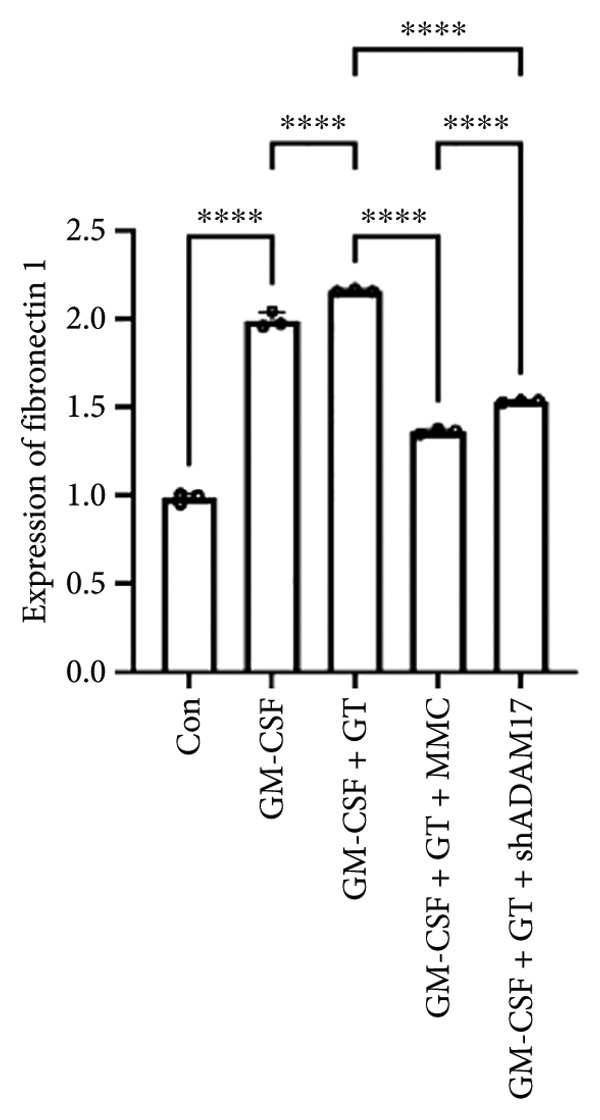
(c)

**Figure:**
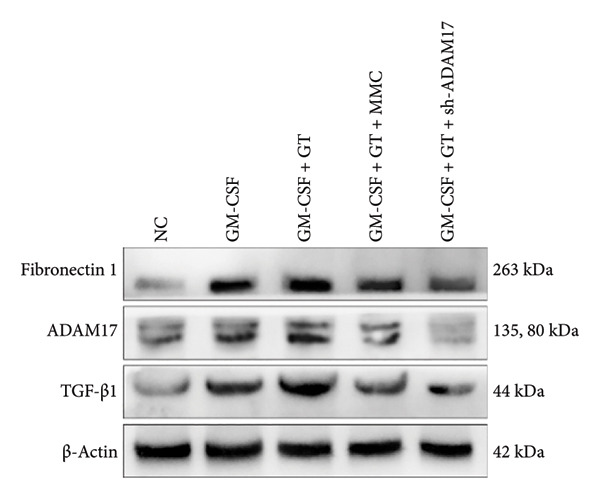
(d)

**Figure:**
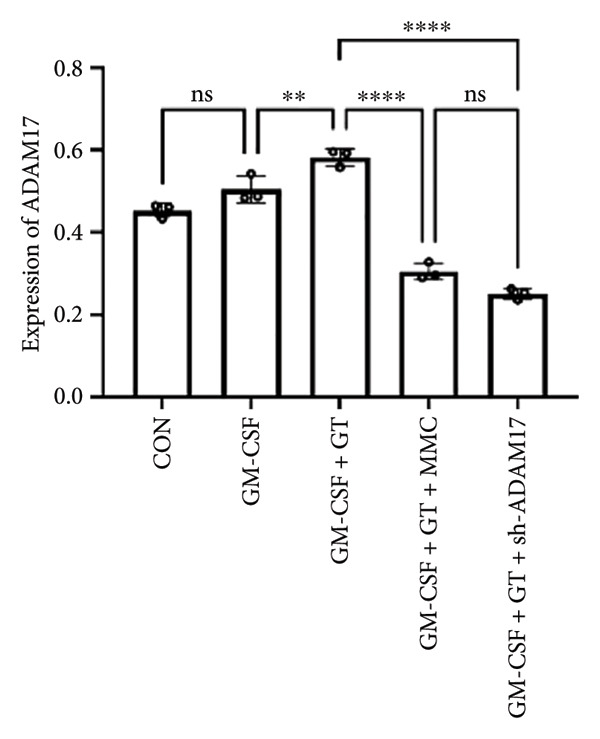
(e)

**Figure:**
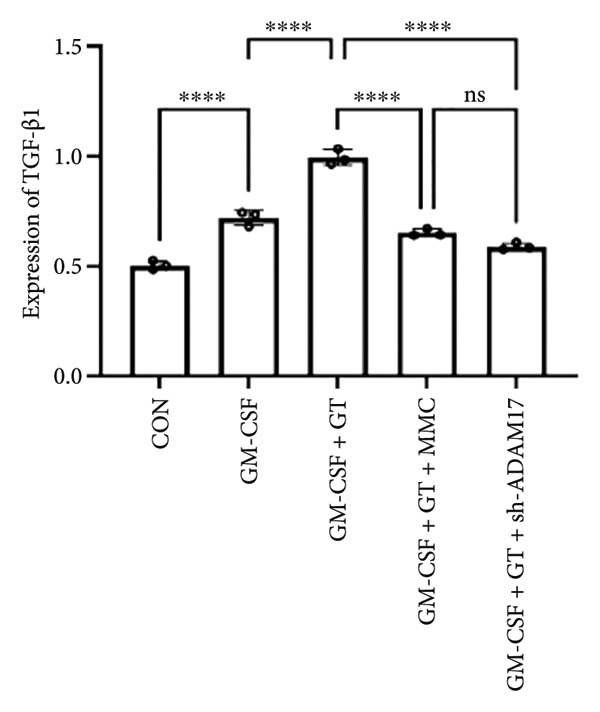
(f)

**Figure:**
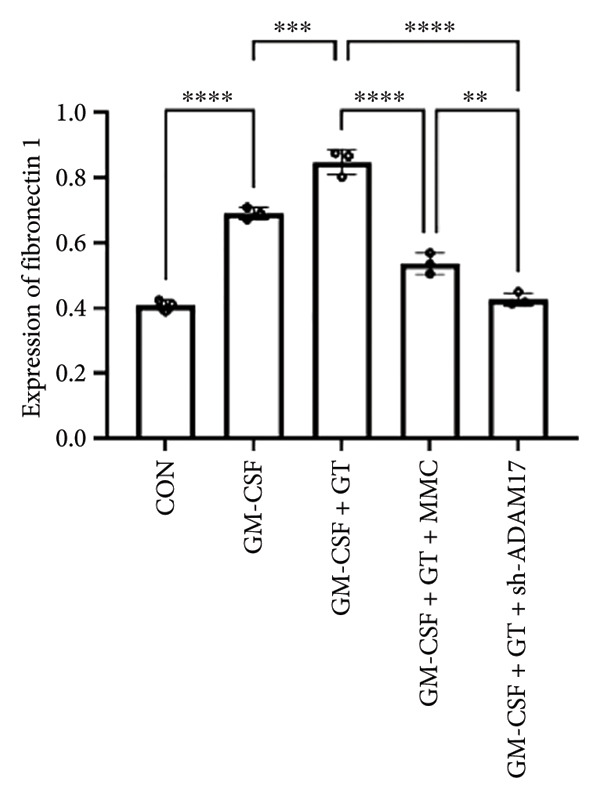
(g)

### 3.4. The Proliferation of Tracheal Epithelial Cells Induced by GM‐CSF Was Inhibited by Lentivirus‐Mediated ADAM17 RNAi

**Figure 6:**
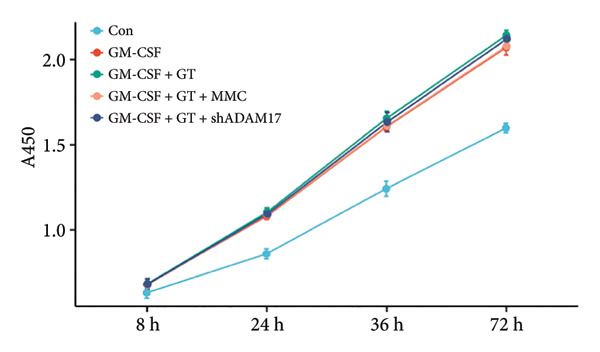
The proliferation of tracheal epithelial cells induced by GM‐CSF was inhibited by lentivirus‐mediated ADAM17 RNAi.

Compared to the control group, the proliferation of tracheal epithelial cells was significantly increased in the induction by GM‐CSF and stimulation with the silicone stent material (*p* < 0.05). Furthermore, lentivirus‐mediated ADAM17 RNAi significantly inhibited the proliferation of tracheal epithelial cells induced by GM‐CSF and stimulated with silicone (*p* < 0.05). Mitomycin also inhibited the proliferation of tracheal epithelial cells induced by GM‐CSF (Figure 6).

## 4. Discussion

TTS is characterized by excessive granulation tissue and scar formation, leading to airway narrowing, which can be life‐threatening [22]. TGF‐β1 has been identified as a key profibrotic factor in airway fibrosis, and its inhibition has been explored as a therapeutic approach for various fibrotic diseases, including idiopathic pulmonary fibrosis and renal fibrosis [23, 24]. TGF‐β1 was shown to alter the expression levels of several fibrosis‐related marker genes and proteins, such as fibronectin 1 [25]. Fibronectin 1 plays a crucial role in various cellular processes, including cell adhesion, migration, proliferation, and wound healing [26]. The expression of fibronectin is closely associated with tissue remodeling during pathological scar formation and tissue repair. The present study showed that lentivirus‐mediated ADAM17 RNAi significantly inhibited the expression of ADAM17, TGF‐β1, and fibronectin 1 induced by GM‐CSF and stimulated with silicone stent material in tracheal epithelial cells. This is consistent with previous studies showing that ADAM17 activates TGF‐β1, thereby promoting fibrotic responses [27]. By targeting ADAM17, TAPI‐1 may disrupt this pathway, thereby lowering the expression of fibronectin 1 and meliorating fibrosis.

In the present study, a severe traumatic airway stenosis model was successfully established in Beagle dogs. HE staining revealed lymphocytic infiltration, proliferation of fibrous tissue, smooth muscle tissue, small blood vessels, and mucosal glands, hyperplasia of the airway epithelium, and thickening of the submucosal layer, leading to lumen narrowing. The present study showed that TAPI‐1 combined with silicone stents significantly alleviated severe TTS in Beagle dogs. These were consistent with decreased the mRNA and protein expressions of ADAM17, TGF‐β1, and fibronectin 1 *in vitro*.

Tracheal stents are commonly used in the treatment of severe TTS. Due to their good tissue compatibility, minimal stimulation of granulation tissue, and strong plasticity, silicone stents are among the most widely used tracheal stents [28]. Although silicone stents are markedly effective in treating severe tracheal stenosis, the granulation tissue growth and restenosis of the trachea cannot be avoided [29]. The present study also indicated silicone stents upregulated ADAM17, TGF‐β1, and fibronectin 1 expression. However, the therapeutic effect of silicone stents is notable in severe TTS. Continuous use of TAPI‐1 before and after the insertion and removal of silicone stents may prevent or reduce granulation tissue growth, thereby preventing tracheal restenosis. Numerous studies have shown that mitomycin can prevent scar formation, excessive repair, and fibrosis, thereby alleviating traumatic airway stenosis [21, 30]. Currently, mitomycin is an invaluable adjunctive treatment for benign airway stenosis [31]. However, there are many side effects of mitomycin including bone marrow suppression, gastrointestinal reactions, local tissue irritation, interstitial pneumonia, and renal impairment [32]. In contrast, TAPI‐1 inhibited the proliferation of tracheal epithelial cells and downregulated the expressions of TGF‐β1 and fibronectin 1 by inhibiting the ADAM17/TGF‐β1 pathway. Moreover, it indicated that the effects of TAPI‐1 were more pronounced than those of mitomycin.

In summary, the present study indicated that TAPI‐1 combined with silicone stents alleviated TTS by inhibiting ADAM17/TGF‐β1 signaling pathway. Furthermore, lentivirus‐mediated ADAM17 RNAi significantly inhibited the proliferation of tracheal epithelial cells induced by GM‐CSF and stimulated by silicone stent material through the ADAM17/TGF‐β1 pathway, which provided a potential therapeutic approach to treat severe TTS.

## Ethics Statement

The Medical Ethics Committee of Hainan General Hospital: Medical Ethics Committee Document No. (2022) 634. The Key Research and Development Project of Hainan Provincial Department of Science and Technology in 2022, entitled “Mechanism Study of 3D‐Printed Drug‐Loaded Stents in Tracheal Reconstruction after Post‐Intubation Tracheal Stenosis” (Principal Investigator: Yaqing Li), has been reviewed and approved by the Hospital’s Medical Ethics Committee. All animal experiments involved in this article comply with relevant medical ethics standards and requirements. The Medical Ethics Committee shall fulfill its duties, adhere to relevant medical ethics regulations and the principle of informed consent for subjects, and ensure that the project is subject to supervision and review from a medical ethics perspective throughout its implementation.

## Disclosure

All the authors reviewed the manuscript.

## Conflicts of Interest

The authors declare no conflicts of interest.

## Author Contributions

Zehua Yang: conceptualization, data curation, formal analysis, investigation, methodology, software, visualization, writing–original draft, and writing–review and editing. Lan Pan: conceptualization, data curation, formal analysis, investigation, methodology, software, visualization, writing–original draft, and writing–review and editing. Tian Xie: editing, data curation, and investigation. Haihong Wu: supervision and validation. Kai Liu: data curation and investigation. Yaqing Li: conceptualization, funding acquisition, resources, supervision, validation, writing–original draft, and writing–review and editing. Zehua Yang and Lan Pan contributed equally to this manuscript.

## Funding

This work was supported by the Hainan Provincial Science and Technology Department Key Research and Development Project under grant number ZDYF2022SHFZ281.

## Data Availability

The data that support the findings of this study are available from the corresponding author upon reasonable request.
